# Improving emergency care in Uganda

**DOI:** 10.2471/BLT.19.020519

**Published:** 2019-05-01

## Abstract

A low-cost emergency care initiative has halved deaths due to emergency conditions in two district hospitals in Uganda. The intervention is being scaled up nationally. Gary Humphreys reports.

Halimah Adam, a nurse at the Mubende regional referral hospital in Uganda, remembers the little boy well. “He was brought into the hospital by his mother,” she says. “He was unconscious and barely breathing.”

The mother told Halimah that the boy had drunk paraffin, mistaking it for a soft drink. Paraffin (kerosene) is poorly absorbed by the gastrointestinal tract, but when aspirated, which can happen when a child vomits, it causes lung inflammation, preventing the lungs from oxygenating the blood. The little boy brought to Halimah had vomit all over his clothes.

Halimah remembers the little boy not because his case was unusual (paraffin ingestion accounts for roughly half of accidental child poisonings in Uganda) but because, for the first time, she knew what to do to save him and had the resources required to do it.

“He was the first person I saved after being trained in the Basic Emergency Care course,” she says. “We placed an oral airway and gave him supplemental oxygen. Two hours later he regained consciousness.”

Around the world, acutely ill and injured people die every day because they lack access to effective, timely prehospital and emergency care. Many countries have no emergency access telephone number to call for an ambulance, and many countries have no ambulances to call. Hospitals lack dedicated emergency units and have few providers trained in the recognition and management of emergency conditions.

“Over half of deaths in low- and middle-income countries are caused by conditions that could be addressed by effective emergency care,” says Dr Teri Reynolds, an expert in emergency, trauma and acute care at the World Health Organization (WHO). “Despite its enormous potential contribution, emergency care has been neglected in health system strengthening. It’s a real blind spot.”

Emergency care systems address a wide range of acute conditions, including those caused by poisonings and other injuries, communicable and noncommunicable diseases, and complications of pregnancy, but they also enhance the impact of other health service interventions by facilitating early recognition of life-threatening conditions and timely access to the right care. “We talk a lot about effectiveness and quality,” says Reynolds, “but we neglect timeliness as a critical component of both. Many ‘proven’ interventions only save lives if given in time in the right situation.”

In Uganda, road traffic crashes are a matter of particular concern. “Uganda has one of the highest incidences of road traffic trauma and deaths on the African continent,” says Joseph Kalanzi, Senior House Officer, Emergency Medicine, Makerere University College of Health Sciences. “We are faced with multiple road traffic crashes daily and have barely any dedicated emergency response.”

According to WHO’s *Global status report on road safety 2018, *road traffic crashes resulted in 12 036 deaths in 2016 in Uganda.

Kalanzi has personal experience of the problem, having dealt with emergency cases when he worked in the Kitovu Hospital in the Masaka District of Uganda in 2010. “People would come in with internal injuries and the best we could do was put them on intravenous fluids and bandage their wounds,” he says. “We did not know how to do anything else, and there was no hope of getting more skills.”

It was partly because of those experiences and a perception that this crucial area was being ignored that Kalanzi committed himself to reforming his country’s emergency care system.

Ugandan health workers are now starting to acquire the skills they need as the result of a low-cost implementation of the WHO emergency care toolkit led by the Ministry of Health (MOH) and supported by WHO and the African Federation for Emergency Medicine, with coordination by Kalanzi. 

The initiative began in 2016 with an assessment of Uganda’s emergency care capacity, using WHO’s Emergency Care System Assessment tool, which helps identify system gaps, and brings together a wide range of stakeholders to come to an agreement on priority actions.

After a meeting convened in Kampala in July 2016, the actions identified included the training and equipping of frontline prehospital and hospital providers, increasing the coverage and quality of ambulance services and improving processes in hospital emergency units. The focus was on the provision of emergency care training for hospital staff, creating dedicated resuscitation areas and basic emergency unit protocols. 

“An intervention was designed based on four WHO tools,” explains Reynolds, “our Basic Emergency Care course, two checklist protocols, a triage protocol, and resuscitation area guidance.” 

The intervention was rolled out in several hospitals, two of which were monitored to assess its impact. The first was Kawolo General Hospital, a 106-bed hospital in the town of Lugazi in the Central Region of Uganda, which handles a significant number of road traffic injuries that occur on the Kampala-Jinja Highway. The second was the above-mentioned Mubende Hospital, a 175-bed regional referral hospital.

Both hospitals had already been gathering baseline data on emergency care outcomes for a year as part of the MOH assessment, and two nurses and a clinical officer had been identified who were willing to put in the effort required to make the necessary changes. 

The initial training of health workers who were eventually to train others took place in the capital, Kampala, but it became clear that better results would be achieved by doing the training on-site. “The training in Kampala was very useful, but it took us away from the hospital where we are needed, and where conditions are not the same as in Kampala anyway,” said nurse Violet Mirembe, who along with Halimah Adam, headed up the initiative at the Mubende Hospital.

“Emergency care has been neglected in health system strengthening. It’s a real blind spot.”

Teri Reynolds.

With no new input of resources, the nurses, supported by their colleagues, adapted the WHO guidance to their settings in several creative ways, including organizing emergency unit beds by triage colour category, and using the course content to create protocol posters and checklists for equipment and supplies. They also broke down the modules of the Basic Emergency Care (BEC) course into daily in-service education sessions and created a “BEC” rapid response team, so that the newly-trained providers could be called to assist anywhere in the hospital. Finally, they used the principles of organizing emergency unit resuscitation rooms to set up “emergency corners” in key inpatient wards.

The results were remarkable. “In the first year of the intervention they roughly halved in-hospital mortality related to emergency conditions,” says Reynolds. “The impact was seen across conditions, ranging from road traffic injury deaths to deaths from pneumonia and diarrhoea in children.”

“What the nurses achieved with the resources they had was extraordinary,” says John-Baptiste Waniaye, Uganda’s Commissioner of Emergency Medical Services in the Ministry of Health, and a major supporter of the emergency service reform initiative.

With the support of the health ministry, the initiative has now been integrated into country-wide reform of the health sector. “The initial plan was to roll out the initiative in five regional hospitals, but a recent commitment of funds as part of the new WHO Global Emergency and Trauma Care Initiative, supported by the Swiss-based AO Foundation, will make it possible to roll it out in all 17 regional hospitals this year,” Waniaye says.

The collaboration of a newly established cadre of Emergency Medical Officers under Waniaye and Kalanzi’s leadership. As part of this reform, emergency care trainings based on the WHO tools used in this pilot will be integrated into the national training curriculum for health workers, including nurses, doctors and students.

Kalanzi celebrates these developments and is keen to emphasize the importance of the health workers who made his vision reality. “The development of systems in which there are no dedicated streams of funding falls on the shoulders of champions,” he says. “So, we need to do everything to nurture and support them, as well as documenting their efforts in a way that can be learned from and modelled.”

WHO launched the Global Emergency and Trauma Care Initiative in December 2018 to support capacity development for the provision of quality emergency care in countries around the world, and to foster awareness through a global advocacy campaign. 

Reynolds welcomes the increased attention and is encouraged by the fact that Member States requested a resolution on emergency care be tabled at the upcoming World Health Assembly. “Our Member States know better than anyone what is needed,” she says. “Their call to action in a high-level forum like the Assembly will have enormous impact.”

**Figure:**
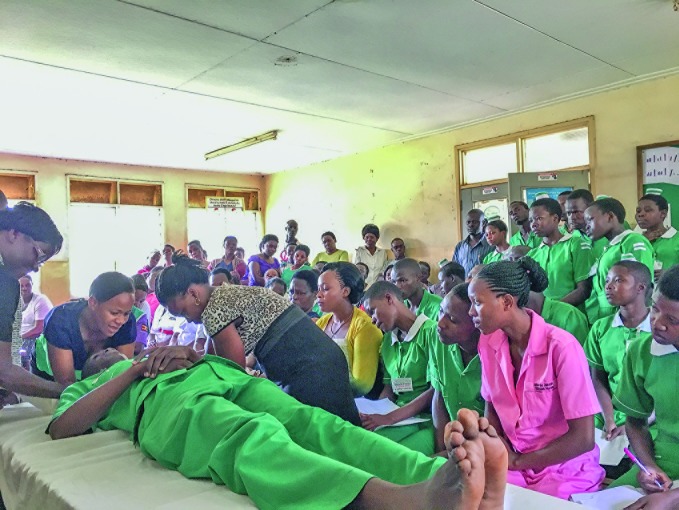
Staff at the Kawolo District Hospital in Lugazi, Uganda learn how to roll a trauma patient as part of their ***Basic emergency care*** training.

Sean Kivlehan/Courtesy of WHO.

**Figure:**
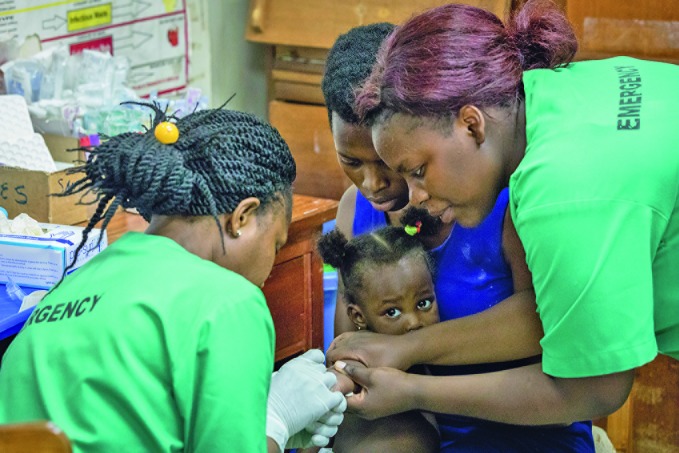
Nurses in the emergency room at Lubaga Hospital, Uganda, install an intravenous line.

Vijay Kannan/Courtesy of WHO

